# Gestational Diabetes Mellitus in Europe: A Systematic Review and Meta-Analysis of Prevalence Studies

**DOI:** 10.3389/fendo.2021.691033

**Published:** 2021-12-09

**Authors:** Marília Silva Paulo, Noor Motea Abdo, Rita Bettencourt-Silva, Rami H. Al-Rifai

**Affiliations:** ^1^ Institute of Public Health, College of Medicine and Health Sciences, United Arab Emirates University, Al Ain, United Arab Emirates; ^2^ Department of Endocrinology and Nutrition, Unidade Local de Saúde do Alto Minho, Viana do Castelo, Portugal; ^3^ Department of Endocrinology, Hospital Lusíadas Porto, Porto, Portugal

**Keywords:** diabetes mellitus, Europe, Gestational Diabetes Mellitus, GDM, systematic review, meta-analysis, pregnancy complications, pregnancy hyperglycemia

## Abstract

**Background:**

Gestational Diabetes Mellitus (GDM) is defined as the type of hyperglycemia diagnosed for the first-time during pregnancy, presenting with intermediate glucose levels between normal levels for pregnancy and glucose levels diagnostic of diabetes in the non-pregnant state. We aimed to systematically review and meta-analyze studies of prevalence of GDM in European countries at regional and sub-regional levels, according to age, trimester, body weight, and GDM diagnostic criteria.

**Methods:**

Systematic search was conducted in five databases to retrieve studies from 2014 to 2019 reporting the prevalence of GDM in Europe. Two authors have independently screened titles and abstracts and full text according to eligibility using Covidence software. A random-effects model was used to quantify weighted GDM prevalence estimates. The National Heart, Lung, and Blood Institute criteria was used to assess the risk of bias.

**Results:**

From the searched databases, 133 research reports were deemed eligible and included in the meta-analysis. The research reports yielded 254 GDM-prevalence studies that tested 15,572,847 pregnant women between 2014 and 2019. The 133 research reports were from 24 countries in Northern Europe (44.4%), Southern Europe (27.1%), Western Europe (24.1%), and Eastern Europe (4.5%). The overall weighted GDM prevalence in the 24 European countries was estimated at 10.9% (95% CI: 10.0–11.8, *I^2^*: 100%). The weighted GDM prevalence was highest in the Eastern Europe (31.5%, 95% CI: 19.8–44.6, *I^2^*: 98.9%), followed by in Southern Europe (12.3%, 95% CI: 10.9–13.9, *I^2^*: 99.6%), Western Europe (10.7%, 95% CI: 9.5–12.0, *I^2^*: 99.9%), and Northern Europe (8.9%, 95% CI: 7.9–10.0, *I^2^*: 100). GDM prevalence was 2.14-fold increased in pregnant women with maternal age ≥30 years (*versus* 15-29 years old), 1.47-fold if the diagnosis was made in the third trimester (*versus* second trimester), and 6.79- fold in obese and 2.29-fold in overweight women (*versus* normal weight).

**Conclusions:**

In Europe, GDM is significant in pregnant women, around 11%, with the highest prevalence in pregnant women of Eastern European countries (31.5%). Findings have implications to guide vigilant public health awareness campaigns about the risk factors associated with developing GDM.

**Systematic Review Registration:**

PROSPERO [https://www.crd.york.ac.uk/PROSPERO/], identifier CRD42020161857.

## Introduction

Hyperglycemia in pregnancy affects about one in every six pregnancies worldwide (1). Gestational Diabetes Mellitus (GDM) is defined as the type of hyperglycemia diagnosed for the first time during pregnancy (2, 3). This has been the widely used definition of GDM for many years, but it presents limitations in terms of the non-possible verification of the preexisting hyperglycemia (4). Hyperglycemia universal routine screening is not available for women at childbearing age before conception or in the first semester, so although GDM can take place at any time during pregnancy, it is more frequently diagnosed after the 24^th^ week of gestation (1, 4).

GDM is highly associated with obesity. Obesity is a growing major public health problem worldwide (5). In 2016, the estimated age-standardized prevalence of obesity and overweight among adult women of the European Region was 24.5% and 54.3%, respectively (6). This prevalence is expected to continue rising in the next years (7, 8). Being overweight (body mass index [BMI] 25.0-29.9 kg/m^2^) or obese (BMI ≥30.0 kg/m^2^) is the most important modifiable risk factor for GDM. The risk is up to 5-fold higher in morbidly obese women, when compared to women with normal body weight (9). Other modifiable risk factors for GDM comprise unhealthy dietary factors, physical inactivity, and cigarette smoking (10). Moreover, the gradual increase in the mean age at childbearing of women in Europe (from 28.8 years in 2013 to 29.3 years in 2018) has an important role in the prevalence of GDM, given that advanced maternal age is a well-known risk factor for GDM (11). The chances of developing GDM increment with previous history of GDM, macrosomia, excessive gestational weight gain, spontaneous abortion, fetal anomalies, preeclampsia, fetal demise, neonatal hypoglycemia, hyperbilirubinemia, and neonatal respiratory distress syndrome family history of type 2 diabetes mellitus (T2DM), polycystic ovary syndrome, parity, non-white ancestry also increment (10, 12).

GDM has potentially serious short- and long-term consequences. The condition is associated with various adverse maternal, fetal, and perinatal outcomes, including but not limited to, preeclampsia, preterm delivery, cesarean section delivery, large for gestational age (LGA) newborns, neonatal hypoglycemia, and Neonatal Intensive Care Unit admission (13). The Hyperglycemia and Adverse Pregnancy Outcomes (HAPO) study reported a continuous association between maternal glucose levels and increased frequency of adverse outcomes, however, there was no obvious threshold at which risk increased (13). Furthermore, the gestational programming and intrauterine fetal exposure to hyperglycemia is an independent risk factor for obesity, hypertension and T2DM in the offspring (14, 15). GDM may play a crucial role in increasing the prevalence of T2DM in women. In the European Region, about 9.6% of women ≥ 25 years old have diabetes (16). A meta-analysis reported a 7-fold increased risk of T2DM in women with GDM compared with those without GDM (17).

Comparing data on GDM is a challenge since there is a lack of universally accepted screening standards and diagnostic criteria. Diagnostic criteria have changed over time and remain controversial, but there has been a move towards the adoption of the International Association of Diabetes in Pregnancy Study Groups (IADPSG) recommendations (18–20). Using the systematic review and meta-analysis approach to understand the regional, sub-regional, and national prevalence of GDM will help the introduction of effective public health measures and enable highlighting the gaps in evidence, following the Guidelines for Accurate and Transparent Health Estimates Reporting (GATHER) (21).

The previously published meta-analysis on the GDM prevalence in Europe was limited to only developed countries in Europe excluding immigrants who did not originate from those developed countries (22). Also, the same meta-analysis was limited to only women tested for GDM in their second or third trimesters (22). To overcome these limitations and provide a more comprehensive and informative assessment on the GDM prevalence in Europe, the present systematic review included all countries in the European region according to the definition of the United Nations (UN) geoscheme and regardless of the original of the included pregnant women. In the present review, the literature search covers a wider range of countries (51 countries) in the European continent regardless of the development status, the origin of the study population, and pregnancy trimester. Moreover, our meta-analyses considered extracting, whenever possible, stratified estimates of the GDM rather than using the overall prevalence reported in the primary studies following a prioritized one-stratification scheme. Indeed, pooling stratified estimates would provide more precise findings on the national, sub-regional, and regional prevalence of GDM. As such, this systematic review and meta-analysis method quantifies the weighted prevalence of GDM in Europe, at regional, sub-regional, and national levels, between 2014 and 2019, according to and regardless of the maternal age, trimester, maternal weight, and GDM diagnostic criteria. It is believed that this study of the 51 countries of the European region regardless of their development will complement the scientific literature, providing more insights into the prevalence of GDM at the subregional level as countries within each subregion in the European continent might have not the same development status interpreted as a limitation in the previous systematic review (22).

## Methods

### Protocol and Registration

We have developed and registered our protocol on PROSPERO (registration number: CRD42020161857). This systematic review and meta-analysis follows the Preferred Reporting Items for Systematic Review and Meta-Analysis (PRISMA) statement (23). The PRISMA checklist is provided elsewhere (see **Supplementary Table S1**).

This systematic review and meta-analysis from prevalence studies in Europe is part of a major study that aims to estimate the prevalence of GDM in different regions in the world. From the same project, the first systematic review and meta-analysis providing findings on the prevalence of GDM in the Middle East and North Africa region has been already completed and submitted for a peer-reviewed journal (24).

### Eligibility Criteria

The search strategy was limited to English language publications between January 2006 and December 2019 and defined in accordance with our population, exposure, comparator, and outcome (PECO) criteria. The population included in this study were all pregnant women tested for GDM during their pregnancy, living in the European region according to the definition of the United Nations (UN) geoscheme (25). All included studies had at least ten pregnant women tested for GDM and reported the prevalence of GDM for their sample or have reported data that allowed us to calculate the GDM prevalence, regardless of the age, trimester, pregnancy status, or GDM ascertainment methodology. However, due to the high number of studies retrieved from databases, we restricted the inclusion criteria to only include studies published between 2014 and 2019.

All studies reporting prevalence estimates on GDM were considered eligible. For this specific systematic review and meta-analysis focusing on the European region, we have excluded studies from the other regions of the globe and studies using unclear GDM diagnostic criteria, unless studies from medical records. These decisions made by the research team were due to the high volume of eligible studies and to produce less potentially biased and more precise estimates on the GDM prevalence.

### Information Sources and Search

A specific search strategy was developed by the principal investigators and a medical librarian expert. The initial search was developed on PubMed-MEDLINE using varied Medical Subject Headings (MeSH) and free-text terms and then translated into EMBASE, Scopus, Web of Sciences, and Cochrane Library, comprising five electronic databases (**Supplementary Table S2**).

### Study Selection

We have used the Covidence software (26, 27) to perform study selection. All citations identified by our search strategy were uploaded into Covidence where duplicates were automatically removed. Two reviewers independently screened the studies for titles and abstracts and subsequently identified potential eligible full-text articles. Conflicts and discrepancies that emerged during the two stages of screening were solved by a third reviewer. The reference lists of eligible studies were also screened to identify additional studies that might have been missed.

### Data Abstraction Process and Data Items

The data we have extracted include the study ID, article type, publication year, journal, country, city, study design, data collection period, population, sample size, sampling strategy, age, pregnancy trimester when GDM was tested, GDM criteria used for diagnosis ascertainment, strata used on the population of the study, the prevalence of GDM in the study sample and by strata whenever available. Furthermore, in research reports presenting more stratified GDM prevalence and at least ten tested subjects per strata, we have extracted the stratified prevalence of GDM following a priority list to avoid double counting: comorbidity, parity, age, pre-gestational BMI, ethnicity, year, placental location, nationality, and occupation. Where there was no stratification on the prevalence of GDM, the overall prevalence was extracted. All relevant data were introduced into a predesigned Excel sheet using string codes and numerical variables. We considered a research report a single publication that might contain data from several studies (each one on a specific population group). In reports where the main study design does not report a clear prevalence, we have extracted the original study design of the report and we have calculated the prevalence of GDM accordingly. In reports where the GDM was ascertained using more than one criterion, the most sensitive and reliable assessment (e.g., fasting glucose blood test *vs*. self-reported) was considered as well as the most recent criteria (e.g., The American Diabetes Association ADA 2010 *vs*. ADA 2006).

### Summary Measures and Synthesis of Results

To estimate the weighted pooled prevalence of GDM and the corresponding 95% confidence interval (CI), we performed meta-analyses of the extracted data. The Freeman–Tukey double arcsine transformation method was applied to stabilize the variances of the prevalence measures (28). The inverse variance method was used to weight the estimated pooled prevalence measures (29). Dersimonian–Laird random-effects model was used to estimate the overall pooled GDM prevalence (30). Cochran’s Q statistic and the inconsistency index I-squared (*I* ^2^), were calculated to measure heterogeneity. Along with the pooled estimates, ranges and median were also reported to describe the dispersion of the GDM prevalence measures reported in the literature. The prediction interval, which estimates the 95% interval in which the true prevalence of GDM in a new study will lie, was also quantified and reported (31).

The overall, country-level and sub-regional levels [Eastern Europe, Northern Europe, Western Europe, and Southern Europe (25)] pooled GDM prevalence was estimated. Moreover, within each sub-European region, the pooled GDM prevalence estimates were generated overall and based on age (<30, ≥30, or unclear age group), pregnancy trimester (first, second, third, or unclear trimester), BMI (normal, overweight, obsess, or unclear BMI), and GDM ascertainment criteria. The provision of pooled estimates regardless of the ascertainment guidelines was justified by the fact that the women were defined and treated as GDM patients following each specific ascertainment guideline. We conducted a synthesis of results including the above-described meta-analysis also comprises a description of the main findings relevant to the study.

### Risk of Bias (RoB)

To test the robustness of the implemented methodology, quality of evidence criteria was also used GDM ascertainment method, sampling methodology, and precision of the estimate. The risk of bias (RoB) tool was performed for each research report and not for individual studies, using the six-quality items adapted from the National Heart, Lung, and Blood Institute (NIH) criteria (32). From the 14 items of the NIH RoB tool we used research question/objective, studied population, participation rate, recruitment, sample size justification, and outcome measures and assessment. Reports were considered to have “high” precision if at least 100 women were tested for GDM. We computed the overall proportion of research reports with potentially low RoB across each of these nine quality criteria and the proportion (out of nine) of quality items with a potentially low RoB for each of the included research reports.

### Publication Bias

The small-study effect on the pooled GDM prevalence estimates was explored through plotting the funnel plot. In the funnel plot, each GDM prevalence measure was plotted against its standard error. The asymmetry of the funnel plot was tested using Egger’s test (33).

Analyses were performed using the *metaprop* (34) and *metareg* packages in Stata/SE v15 (35).

## Results

### Study Selection

**Figure 1:**
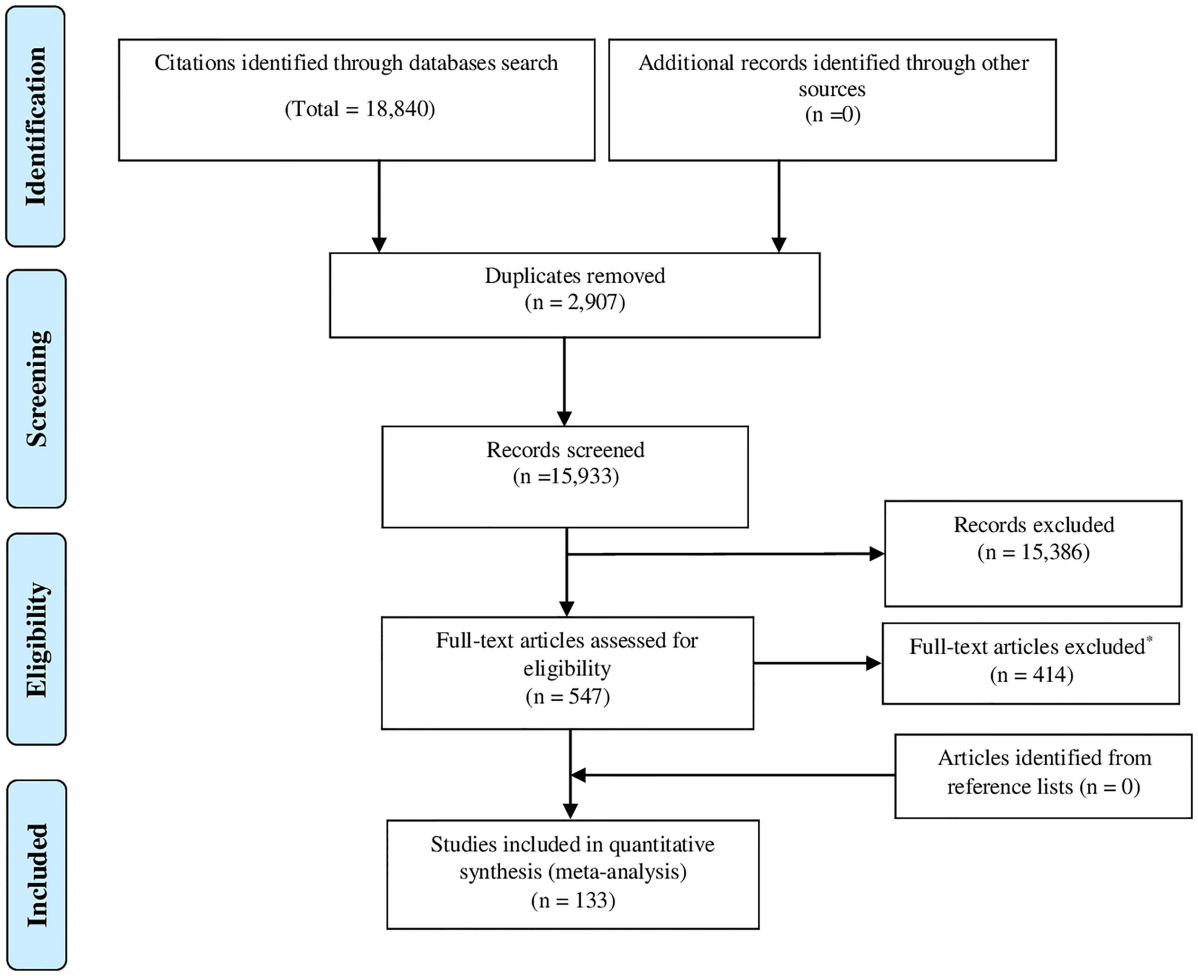
PRISMA flowchart. • Reasons for full-text exclusion: 214 GDM or DM total population 78 Wrong setting 29 Not in Europe 24 GDM Prevalence was incalculable 23 reported an unclear ascertainment of GDM criteria (I report containing information front Albania, 3 from Denmark, 2 from Finland, 2 from Ireland, 5 front Italy, I from Netherlands, 2 f:om Poland, 2 from Portugal, 2 from Spain, and 3 from United Kingdom). 15 reports have duplicate data [I from Croatia (30), I from France (31), I from Italy (32), 2 from Netherlands (33,34),6 from Norway (35-40), and 4 from United Kingdom (41-44)], and only the report that first published the study data was used. 9 Conference abstract with not enough information 8 Case-control (GDM *vs*. non-GDM) 7 Duplicates 6 Wrong patient population 1 Year of GDM diagnosis is UNCLEAR (Not mentioned).

After de-duplication, 15,933 records were screened and 547 full-text research reports critically assessed for eligibility, 133 research reports were deemed eligible and included in the meta-analysis (**Figure 1**).

### Study Characteristics

The 133 research reports related to 24 countries in Europe and tested a total of 15,572,847 pregnant women for GDM and yielded 254 GDM prevalence studies. The majority of the research reports were reported from Northern Europe (59/133), followed by Southern Europe (36/133), Western Europe (32/133), and Eastern Europe (6/133). Across the four UN geoscheme sub-regions (25) the most studied countries were Italy (21 reports) and the United Kingdom (14 reports). **Tables 1**–**4** summarize basic characteristics of the included research articles in the four European sub-regions.

#### Eastern Europe

From the Eastern Europe countries (Belarus, Bulgaria, Czech Republic, Hungary, Poland, Republic of Moldova, Romania, Russian Federation, Slovakia, and Ukraine), our search has just captured six reports that tested a total of 12,122 pregnant women for GDM from Hungary (two reports), Poland (three reports), and Republic of Macedonia (one report). In two out of the eight GDM prevalence studies reported in these three countries, GDM ascertainment was based on the Polish Gynecological Society Guidelines (**Table 1**).

**Table 1:** Baseline studies characteristics from Eastern Europe.

Author (Ref)	Duration of data collection	City	Sampling strategy	Population	Ascertainment method	Tested sample	GDM Positive	Prev. (%)
Hungary								
Renes L. et al. (36)	01/2014 – 12/2014	Hungary, Szeged	Consecutive	General population	WHO 1999	1493	155	10.1%
Kun A. et al. (37)	01/2009 – 12/2017	Hungary (Western)	Consecutive	General population	WHO 2013	9469	1505	14.9%
Poland								
Mac-Marcjanek K. et al. (38)	06-2011 – 06/2013	Poland, Lodz	Unclear	Caucasian pregnant women	PDA 2011	145	113	78%
					PDA 2014		104	71.7%
Kosinska-Kaczynska K. et al. (39)	01/2007 – 06/2016	Poland, Warsaw	Unclear	Women with dichorionic twin pregnancies at <14 weeks of pregnancy	Polish Gynaecological Society Guidelines	201	27	13.4%
Szymusik I. et al. (40)	07/2013 – 12/2016	Poland, Warsaw	Consecutive	General population	Polish Gynaecological Society Guidelines	368	31	8%
Republic of Moldova								
Brankica K. et al. (41)	01/2013 – 06/2013	Republic of Moldova, Skopje	Consecutive	General population	IADPSG	118	78	66.1%

IADPSG, International Association of Diabetes in Pregnancy Studies Group; PDA, Polish Diabetes Association; WHO, World Health Organization.

#### Northern Europe

From Northern Europe sub-region (Denmark, Estonia, Finland, Iceland, Ireland, Latvia, Lithuania, Norway, Sweden, and United Kingdom), there were 59 reports presenting estimates on GDM prevalence. None of those reports were from Estonia or Latvia. Seven reports reporting 17 GDM prevalence studies were from Denmark, 10 reports with 22 GDM prevalence studies were from Finland, one report with three GDM prevalence studies were from Iceland, seven reports with 10 GDM prevalence studies were from Ireland, two reports with three GDM prevalence studies were from Lithuania, nine reports with 19 GDM prevalence were studies from Norway, nine reports with 20 GDM prevalence were studies from Sweden and 14 reports with 28 GDM prevalence studies were from the United Kingdom. In the 122 GDM prevalence studies that tested a total of 10,278,921 pregnant women reported in the Northern European countries, the IADPSG (in 15 out of 122 studies) followed by the WHO 2013 (in 14 out of 122 studies) were the most common used GDM diagnostic (**Table 2**).

**Table 2:** Baseline studies characteristics from Northern Europe.

Author (Ref)	Duration of data collection	City	Sampling strategy	Population	Ascertainment method	Tested sample	GDM Positive	Prev. (%)
Denmark								
Bonnesen B. et al. (42)	01/2009-12/2010	Denmark, Hvidovre	Consecutive	Primiparous women with a spontaneous singleton pregnancy	Medical Records	3,440	43	1.3%
Medek H. et al. (43)	05/2012 – 10/2013	Denmark, Reykjavik	Consecutive	General population	IADPSG	117	22	12.4%
Holst S. et al. (44)	01/2006 – 12/2010	Denmark, National	Whole population	Women with singleton pregnancies	Medical Records	264539	5781	2.2%
Jeppesen C. et al. (45)	01/2012 – 12/2012	Denmark, National	Whole population	Women aged 15-49 years old	Medical Records	56894	1721	3.0%
McIntyre HD. et al. (10)	01/2010 –12/2012	Denmark, National	Whole population	General population	WHO 2013	1516	620	40.1%
Hamann CR. et al. (46)	01/1997 –12/2014	Denmark, National	Whole population	General population	Medical Records	104410	1914	1.8%
				Women with atopic dermatitis any time prior to birth		10441	175	1.7%
				Women with atopic dermatitis 24 months prior to birth		1064	15	1.4%
				Women with atopic dermatitis during pregnancy		319	3	0.9%
Strand-Horlm KM. et al. (47)	01/2004 – 12/2012	Denmark, Aarhus	Whole population	Women with singleton pregnancies	WHO 2013	42571	928	2.2%
Finland								
Koivusalo SB. et al. (48)	01/2008 – 12/2014	Finland, Lappeenranta	Random selection	Women with a history of GDM or pre-pregnancy obesity	ADA 2007	269	47	17.4%
Ellenberg A. et al. (49)	01/2006 – 12/2008	Finland, National	Whole population	Women with singleton pregnancies	Medical records	34460	2522	7.2%
	01/2010 –12/2012					36331	4128	11.3%
Koivunen S. et al. (50)	01/2006 – 12/2006	Finland, National	Consecutive	Pregnant at gestational age ≥ 22 weeks or a birthweight ≥ 500 g	The Finnish Current Care guidelines	15682	5179	9.1%
	01/2012 – 12/2010					30365	6679	11.3%
Meinilä J. et al. (51)	01/2008 – 12/2014	Finland, Helsinki Metropolitan area and Lappeenranta	Unclear	Women at high risk of GDM due to obesity, history of GDM, or both	ADA 2008	251	46	18.3%
Laine MK. et al. (52)	01/2009 – 12/2015	Finland, Vantaa	Whole population	Primiparous women	The Finnish Current Care guidelines	7750	1281	16.5%
Laine MK. et al. (53)	01/2009 – 12/2015	Finland, Vantaa	Whole population	Primiparous women with height < 159 cm	The Finnish Current Care guidelines	689	198	28.7%
				Primiparous women Primiparous women with height between 164-167 cm		1221	243	19.9%
Girchenko P. et al. (54)	01/2011 – 12/2012	Finland, National	Whole population	General population	Medical records	2504	248	9.9%
Kong L. et al. (55)	01/2004 – 12/2014	Finland, National	Whole population	General population	Medical records	649043	98568	15.2%
Ellfolk M. et al. (56)	01/1996 – 12/2016	Finland, National	Whole population	Women exposed to antipsychotics	Medical records	21125	3047	14.4%
Ijas H. et al. (57)	01/2009 – 12/2009	Finland, National	Whole population	Women with singleton pregnancies	Medical records	24555	5658	23.4%
Iceland								
Tryggvadottir EA. et al. (58)	04/2012 – 10/2013	Iceland, Reykjavik	Consecutive	Non-smoking women and without GDM risk factors	WHO 2013	168	17	10.1%
Ireland								
Lindsay KL. et al. (59)	03/2012 – 03/2013	Ireland, Dublin	Random sampling	Obese women	Carpenter and Coustin	138	6	4.3%
Daly N. et al. (60)	04/2014 – 08/2014	Ireland, Dublin	Convenience	Obese European women	WHO 2013	24	16	66.7%
Mone F. et al. (61)	01/2011 – 09/2012	Ireland, Dublin	Whole population	General population	WHO 2013	7252	140	1.9%
Moore R. et al. (62)	2007 -2013	Ireland, Dublin	Unclear	HIV women	Carpenter and Coustin	142	3	2.1%
O’Dea A. et al. (63)	01/2013 – 12/2013	Ireland, Galway	Convenience	General population	IADPSG	690	48	7.0%
Farren N. et al. (64)	01/2014 – 01/2016	Ireland, Dublin	Consecutive	Women with family history of DM	IADPSG	240	40	16.6%
Daly N. et al. (65)	11/2013 – 04/2016	Ireland, Dublin	Consecutive	Women with BMI ≥ 30 that participated in the intervention	IADPSG	43	25	58.1%
				Women with BMI ≥ 30 that did not participate in the intervention		43	21	48.8%
Lithuania								
Ramoniene G. et al. (66)	01/2010 – 12/2010	Lithuania, Khaunas	Consecutive	Obese women with singletons	WHO 1999	140	33	23.6%
				Normal weight women with singletons		3107	160	5.1%
Malakauskiene L. et al. (67)	01/2005 – 12/2015	Lithuania, National	Whole population	Pregnant after bariatric surgery	Medical records	130	3	2.31%
Norway								
Rasmussen S. et al. (68)	01/2007 – 12/2010	Norway, National	Whole population	General population	Medical records	77294	1086	1.4%
Sommer C. et al. (69)	05/2008 – 05/2010	Norway, Oslo	Unclear	General population	IADPSG	728	229	31.5%
Helseth R. et al. (70)	04/2007 – 06/2009	Norway, Trondheim, and Stavanger	Unclear	Nordic Caucasian women	WHO 2013	687	42	6.1%
Leirgul E. et al. (71)	01/2006 – 12/2009	Norway, National	Whole population	General population	Medical records	233303	3484	1.5%
Garnæs KK. et al. (72)	11/2012 – 03/2013	Norway, Trondheim	Unclear	Women with BMI ≥ 28 that participated in the intervention	WHO 2013	46	8	18.2%
				Women with BMI ≥ 28 that did not participate in the intervention		45	13	29.5%
Sorbye LM. et al. (73)	01/2006 – 12/2014	Norway, National	Whole population	Women in their second pregnancy	Norwegian Society of Gynecology and Obstetrics	24198	439	1.8%
Lehmann S. et al. (74)	01/1967 – 12/2014	Norway, National	Whole population	Women who trial labor after caesarean section	Medical records	1119	686	63.0%
Sole KB. Et al. (75)	01/1999 – 12/2014	Norway, National	Whole population	Women with singleton pregnancies	Medical records	907048	14200	1.57%
Magnus MA. et al. (76)	01/2009 – 12/2013	Norway, National	Whole population	General population	Medical records	162343	5938	3.7%
Sweden								
Lindqvist M. et al. (77)	2011 – 2012	Sweden, National	Whole population	General population	Medical records	181292	2548	1.4%
Nilsson C. et al. (78)	2012 – 2013	Sweden, National	Whole population	General population	WHO 1999	7491	210	2.8%
Stokkeland K. et al. (79)	2006 – 2011	Sweden, National	Whole population	General population	Medical records	576642	6343	1.0%
Sundelin HEK. et al. (80)	2006 – 2014	Sweden, National	Whole population	General population	Medical records	877742	9919	1.1%
Stogianni A. et al. (81)	2009 – 2012	Sweden, Kronoberg	Whole population	General population	Medical records	280	97	34.6%
Crump C. et al. (82)	1973 - 2014	Sweden, National	Whole population	General population	Medical records	4186615	34255	0.8%
Hilden K. et al. (83)	1998 – 2012	Sweden, National	Whole population	General population	Medical records	1294006	14833	1.0%
Khashan AS. et al. (84)	1982 – 2012	Sweden, National	Whole population	General population	Medical records	1292792	4967	0.4%
Liu C. et al. (85)	2014 – 2017	Sweden, National	Whole population	Refugees	Medical records	31897	1148	3.6%
United Kingdom								
Farrar D. et al. (86)	2008 – 2010	UK, Bradford	Whole population	General population	WHO 1999	11516	1132	10%
West J et al. (87)	03/2007 – 12/2010	UK, Bradford	Consecutive	Caucasian British/Irish women	WHO 1999	3503	172	4.9%
				Pakistani women		2656	406	15.3%
Syngelaki A. et al. (88)	03/2006 – 07/2013	UK, London and Gillingham	Unclear	General population	Mixed methods	75161	1827	2.4%
Poston L. et al. (89)	03/2009 – 06/2014	UK, London, Bradford, Glasgow, Manchester, Newcastle, Sunderland	Random sampling	Obese women	IADPSG	1280	332	26%
Sovio U. et al. (90)	08/2008 – 07/2012	UK, Cambridge	Unclear	Nulliparous women	Mixed methods	4069	171	4.2%
Murphy NM. et al. (91)	05/2007 – 02/2011	UK, London, Manchester, Cork, Leeds	Unclear	Women at high risk of GDM	Mixed methods	395	35	8.9%
				Pregnant women without risk of GDM		261	20	7.7%
White SL. et al. (92)	2009 – 2014	UK	Unclear	Obese women	IADPSG	1303	337	25.9%
Hanna FW. et al. (93)	02/2010 – 12/2013	UK	Unclear	General population	NICE 2015	6930	967	13.7%
Panaitescu AM. et al. (94)	03/2006 – 11/2015	UK, London	Unclear	General population	WHO 1999	107788	2542	2.4%
Hall E. et al., (95)	05/2017 – 08/2017	UK, London	Whole population	General population	NICE 2015	1267	264	21%
Balani J. et al. (96)	2010 – 2011	UK, Surrey	Unclear	Obese women	WHO 1999	302	72	23.8%
Nzelu D. et al. (97)	2011 – 2016	UK, London	Consecutive	Pregnant women with pregnancy induced hypertension	NICE 2015	773	93	12%
Vieira MC. et al. (98)	03/2009 – 06/2014	UK, London	Whole population	Obese women	IADPSG	824	241	29.6%
Wagnild JM. et al. (99)	02/2017 – 08/2017	UK, Northeast England	Consecutive	Women at high risk of GDM	NICE 2015	326	31	16.5%

ADA, American Diabetes Association; BMI, body mass-index; DM, diabetes mellitus; GDM, gestational diabetes mellitus; IADPSG, International Association of the Diabetes and Pregnancy Study Groups; NICE, National Institute for Health and Care Excellence; UK, United Kingdom; WHO, World Health Organization.

#### Western Europe

From Western Europe sub-region (Austria, Belgium, France, Germany, Liechtenstein, Luxembourg, Monaco, Netherlands, and Switzerland). In this sub-region, the majority of the 32 research reports were in France (34.4%) followed by Germany (18.8%), Austria (15.6%), and Switzerland (15.6%). Our study did not find any prevalence studies on GDM from three countries (Liechtenstein, Luxembourg, and Monaco) in this sub-region reported between 2014 and 2019. In the 55 GDM prevalence studies that tested a total of 4,212,723 pregnant women in the Western European countries, the IADPSG (in 14 studies) was the most commonly used GDM diagnostic (**Table 3**).

**Table 3:** Baseline studies characteristics from Western Europe.

Author (Ref)	Duration of data collection	City	Sampling strategy	Population	Ascertainment method	Tested sample	GDM Positive	Prev. (%)
Austria								
Bozkurt L. et al. (100)	2010 – 2014	Austria, Vienna	Unclear	General population with OGTT at 16 weeks	IADPSG	221	81	38.3%
Tramontana A. et al. (101)	01/2010- 11/2013	Austria, Vienna	Whole population	General population	IADPSG	4948	209	4.2%
Tramontana A. et al. (102)		Austria, Vienna	Whole population	Women with high-risk pregnancies	IADPSG	382	170	44.5%
Koninger A. et al. (103)	2009 – 2018	Austria, Essen	Whole population	Women with polycystic ovary syndrome	GDDD	63	29	46%
Weiss C. et al. (104)	01/2013 – 12/2015	Austria, Linz	Whole population	Singleton pregnancies	WHO 2013	3293	553	16.8%
Belgium								
Benhalima K. et al. (105)	01/2010 – 12/2013	Belgium, Leuven, Aalst	Whole population	General population	Carpenter-Coustan	14661	601	4.1%
De Munck N. et al. (106)	03/2010 – 08/2014	Belgium, Brussels	Whole population	Ocyte recipient with use of closed vitrification	Mix method	112	13	11.6%
France								
Grunewald D. et al. (107)	2008-2013	France, Paris	Unclear	Pregnant women with cystic fibrosis	Medical records	23	2	8.7%
Miailhe G. et al. (108)	04/2011 – 02/2012	France, Paris	Whole population	Singleton pregnancies	IADPSG	2187	309	14%
Goueslard K. et al. (109)	2007 – 2013	France, National	Whole population	General population	Medical records	1515387	62958	4.14%
Regnault N. et al. (110)	2013	France, Bondy	Whole population	General population	Medical records	788494	67810	8.6%
Mortier I. et al. (111)	01/2011 – 07/2012	France, Marseille	Whole population	Singleton pregnancies	IADPSG	444	60	13.5%
Boudet-Berquier J. et al. (112)	01/2012 – 04/2014	France, National	Random sampling	General population	Mixed methods	3204	247	7.7%
Billionnet C. et al. (113)	2012	France, National	Whole population	General population	Medical records	796346	57629	7.24%
Mitanchez D. et al. (114)	08/2010 – 03/2013	France, Paris	Unclear	Singleton pregnancy in obese women	IADPSG	226	99	43.8%
				Singleton pregnancy in normal weight women		222	41	18.4%
Marie C. et al. (115)	2006	Auvergne, France	Whole population	General population	Carpenter-Coustan	1175	73	6.2%
	2010					2840	156	5.5%
Preaubert L. et al. (116)	01/2010 – 12/2016	France, Paris	Whole population	Ocyte recipient with use of closed vitrification	IADPSG	247	39	15.8%
Soomro MH. et al. (117)	03/2003 – 01/2006	France, Poitiers and Nancy	Whole population	Women with blood-biomarkers to study heavy metals	Carpenter-Coustan	623	4	7.1%
Germany								
Stuber TN. et al. (118)	2006 – 2011	Germany, Wurzburg	Whole population	General population	Medical records	2810	264	9.4%
Beyerlein A. et al. (119)	2008 – 2014	Germany, Bavaria	Consecutive	General population	Medical records	173718	6427	3.7%
Tamayo T. et al. (120)	07/2012 – 06/2013	Germany, North Rhine	Consecutive	General population	IADPSG	153302	9229	6.0%
	07/2013 – 06/2014					158839	10817	6.8%
Melchior H. et al. (121)	01/2014 – 12/2015	Germany, National	Whole population	General population	Medical records	567191	74869	13.2%
Köninger A. et al. (122)	2014 -2016	Germany, Essen	Unclear	Singleton pregnancies	German Diabetes Association	105	29	27.64
Pahlitzsch TMJ. et al. (123)	2001 – 2017	Germany, Solingen	Whole population	Mothers of macrocosmic newborns	Medical records	2277	87	3.8%
Netherlands								
Lamain-de-Ruiter ML (124).	12/2010 – 01/2014	Netherlands	Unclear	General population	Mixed method	3723	181	4.9%
Koning SH. et al. (125)	01/2011 – 09/2016	Netherlands, Groningen	Whole population	Pregnant women with at least one risk factors for GDM	WHO 2013	10642	3364	31.6%
De Wilde MA. et al. (126)	04/2008 – 04/2012	Netherlands	Unclear	Women with polycystic ovarian syndrome	ADA 2004	188	43	23.%
	12/2012 – 12/2013			Singleton pregnancies	WHO 1999	2889	129	4.5%
Switzerland								
Mosimann B. et al. (127)	01/2014 – 12/2014	Switzerland, Bern	Consecutive	General population	Mixed method	328	51	15.5%
Amylidi S. et al. (128)	06/2011 – 11/2012	Switzerland, Bern	Whole population	Pregnant women with at least one risk factors for GDM	ADA 2016	218	32	14.7%
Ryser Rüetschi J. et al. (129)	10/2010 – 04/2012	Switzerland, Geneva and Basel	Consecutive	General population	IADSPG	2298	251	10.9%
Horsch A. et al. (130)	11/2012 * 07/2013	Switzerland, Lausanne	Whole population	General population	Mixed method	203	39	19.2%
Savopol H. et al. (131)	01/2014 – 12/2015		Whole population	General population	IADSPG	502	159	31.7%

ADA, American Diabetes Association; DM, diabetes mellitus; GDDD, Deutsche Gesellschaft fur gynakologie und Geburtshilfe; HBV, Hepatitis B virus, HIV, Human Immunodeficiency virus; IADPSG, International Association of the Diabetes and Pregnancy Study Groups; OGTT, oral glucose tolerance test; WHO, World Health Organization.

#### Southern Europe

From Southern Europe sub-region (Albania, Andorra, Bosnia and Herzegovina, Croatia, Greece, Italy, Malta, Montenegro, North Macedonia, Portugal, San Marino, Serbia, Slovenia, and Spain), there were 36 research reports, of which, the majority were from Italy (58.3%) followed by 19.4% were from Spain. Between 2014 and 2019, there were no prevalence studies on GDM from Albania, Andorra, Bosnia and Herzegovina, Montenegro, North Macedonia, Portugal, San Marino, and Serbia. In the 69 GDM prevalence studies that tested a total of 1,069,081 pregnant women, the IADPSG was the most common GDM ascertainment criteria used (30.4%) (**Table 4**).

**Table 4:** Baseline studies characteristics from Southern Europe.

Author (Ref)	Duration of data collection	City	Sampling strategy	Population	Ascertainment method	Tested sample	GDM Positive	Prev. (%)
Croatia								
Djakovicí I. et al. (132)	2011 – 2012	Croatia, Zagreb	Consecutive	General population	HAPO study guidelines	6407	593	9.3%
Djelmis J. et al. (133)	2012 -2014	Croatia	Unclear	Singleton pregnancies	IADSPG	4646	1074	23.1%
					NICE 2015	4646	826	17.8%
Erjavec K. et al. (134)	2010	Croatia, National	Consecutive	General population	WHO 1999	42656	953	2.2%
	2014				IADSPG	39092	1829	4.6%
Vince K, et al. (135)	2011	Croatia, National	Consecutive	General population	IADSPG	40641	1181	2.9%
Cyprus								
Inancli SS et al. (136)	11/2013 – 04/2014	Cyprus, National	Consecutive	Turkish Cypriot	National Diabetes Data Group	230	45	19.6%
Greece								
Vassilaki M. et al. (137)	02/2007 – 02/2008	Greece, Crete	Convenience	General population	Carpenter-Coustan	1122	102	9.1%
Italy								
Trotta F. et al. (138)	10/2009 – 09/2010	Italy, Lombardy	Whole population	General population	Medical records	86171	1921	2.3%
Pintaudi B. et al. (139)	05/2010 – 10/2011	Italy, Messina	Consecutive	Caucasian women	IADSPG	1015	113	11.1%
Caserta D. et al. (140)	01/2007 – 06/2011	Italy, Rome	Whole population	Twin pregnancies	Medical records	207	6	2.9%
				Twin pregnancies with assisted conception		138	14	10.1%
Lacaria E. et al. (141)	01/2012 – 13/2013	Italy, Pisa and Livorno	Consecutive	Caucasian women	IADSPG	2497	279	11.1%
D’Anna R. et al. (142)	01/2011 – 04/2014	Italy, Messina and Modina	Random sampling	Obese women	IADSPG	241	51	23.8%
Pinzauti S. et al. (143)	01/2010 – 12/2014	Italy, Florence, and Siena	Whole population	Twin pregnancies with assisted conception	Mixed method	430	30	6.9%
Capula C. et al. (144)	08/2011 – 01/2015	Italy, Catanzaro	Convenience	Healthy pre-pregnancy women	IADSPG	3974	1066	26.8%
Santamaria A. et al. (145)	01/2012 – 12/2014	Italy, Messina and Modena	Convenience	Overweight Caucasian	ADA 2011	102	28	27.5%
				Overweight Caucasian receiving Myo-inositol		95	11	11.6%
Bianchi C. et al. (146)	01/2010 – 03/2015	Italy, Pisa	Unclear	General population	Medical records	1198	476	39.7%
Di Cianni G. et al. (147)	01/2015 – 12/2015	Italy, Tuscany	Whole population	General population	Medical records	17606	2000	11.4%
Bordi et al. (148)	01/2001 – 06/2015	Italy, Rome	Whole population	Twin pregnancies with assisted conception	Medical records	450	38	8.4%
				Twin pregnancies		647	18	2.8%
Chiefari E. et al. (149)	08/2011 – 12/2016	Italy, Cantazaro	Unclear	General population	Italian Minister Guidelines	5473	1559	28.5%
Cozzolino M, et al. (150)	01/2010 – 01/2016	Italy, Florence	Whole population	Multiple pregnancies	IADSPG	656	99	15.1%
Bruno R. et al. (151)	02/2013 – 06/2014	Italy, Modena	Unclear	Singleton pregnancies of overweight/obese women with prescribed personalized dietary intervention	IADSPG	62	23	37.1%
				Singleton pregnancies of obese women		69	13	18.8%
Bianchi C. et al. (152)	01/2013 – 12/2015	Italy, Pisa	Whole population	General population	Italian National Guidelines	1338	534	39.95
Meregaglia M. et al. (153)	01/2014 – 12/2014	Italy, National	Whole population	General population	Medical records	44402	11540	10.9%
Quaresima P. et al. (154)	01/2015 – 12/2016	Italy, Catanzaro	Consecutive	General population	IADSPG	1413	451	31.8%
Gerli S. et al. (155)	01/2011 – 12/2013	Italy, National	Whole population	Women in Robson class 1 according to the Ten Group Classification System	IADSPG	7693	132	1.7%
				Women in Robson class 3 according to the Ten Group Classification System		4919	95	1.9%
Masturzo B. et al. (156)	01/2011 – 12/2015	Italy, Turin	Whole population	Singleton pregnancies	Medical records	27807	2308	8.3%
Visconti F. et al. (157)	08/2011 – 12/2016	Italy, Calabria	Consecutive	Singleton pregnancies	IADSPG	2424	596	24.7%
Marozio L. et al. (158)	2009 - 2015	Italy, Turin	Whole population	Pregnant women < 40 years old	ADA 2014	52413	1430	2.7%
				Pregnant women between 40-44 years old		3541	203	5.7%
				Pregnant women > 45 years old		257	21	8.2%
Malta								
Xuereb S. et al. (159)	01/2009 – 12/2009	Malta, National	Consecutive	General population	WHO 2006	203	43	21.2%
Slovenia								
Kek T. et al. (160)	05/2013 – 09/2015	Slovenia, Ljubljana	Unclear	General population	Self-reported	450	43	10.0%
Spain								
Goni L. et al. (161)	11/2009 – 03/2010	Spain, Navarra	Convenience	General population	Medical records	5987	397	7.8%
Ruiz-Gracia T. et al. (162)	04/2011 – 03/2012	Spain, Madrid	Consecutive	General population	Carpenter-Coustan	1750	185	10.5%
Berglund SK. et al. (163)	2008 - 2012	Spain, Granada	Convenience	Overweight and Obese women	Spanish Society of Gynecology and Obstetrics	333	46	13.8%
Benaiges D. et al. (164)	04/2013 – 09/2015	Spain, Barcelona	Consecutive	Singleton pregnancies	National Diabetes Data Group	1158	152	13.1%
Assaf-Balut C. et al. (165)	01/2015 – 12/2015	Spain, Madrid	Consecutive	Single pregnancy following standard Med-Diet supplemented with EVOO and pistachios	IADSPG	434	74	17.1%
				Single pregnancy following standard Med-Diet		440	74	23.4%
Gortazar L. et al. (166)	2006 – 2015	Spain, Catalonia	Whole population	Singleton pregnancies	Medical records	739877	35729	4.8%
Mane L. et al. (167)	2010 - 2013	Spain, Barcelona	Whole population	General population	Self-reported	5633	572	10%

ADA, American Diabetes Association; EVOO, extra virgin olive oil; HAPO, Hyperglycemia and Adverse Pregnancy Outcomes; IADPSG, International Association of the Diabetes and Pregnancy Study Groups; WHO, World Health Organization.

### Weighted GDM Prevalence

In the 15,572,847 pregnant women tested for GDM the weighted GDM prevalence estimated was 10.9% (95% CI: 10.0–11.8%, *I^2^*, 100%) in the 24 countries out of a total of 48 countries in Europe. Of the tested pregnant women, 76.6% were from three countries: Sweden (48%), France (20.0%), and Norway (8.6%). From the represented countries in our analysis, Sweden (Northern Europe) shows the lowest weighted GDM prevalence of 1.8% (95% CI: 1.5–2.2, *I^2^*, 99.9%) (**Table 5**). The highest observed national-based prevalence of 66.1% from a single study in the Republic of Moldova has contributed to the observed highest weighted GDM prevalence in the Eastern Europe sub-region (**Table 5**).

**Table 5:** Weighted national, sub–regional, and regional GDM prevalence in Europe.

Country	No. of studies	Tested sample	GDM	GDM prevalence				Heterogeneity measures			
				Range (%)	Median(%)	Weighted prev. %	95% CI	Q (*p*−value)^1^	*I^2^* (%)^2^	95% PI (%)^3^	*P*–value^4^(fixed)
***Eastern Europe***											p<0.001 (p<0.001)
Hungary	2	10,962	1,660	10.1–14.9	12.5	15.1	14.4–15.8	–	–	–	
Poland	5	1,042	298	8.0–78.0	13.4	34.1	8.8– 65.8	427.8 (p<0.001)	99.1	0.00–100	
Republic of Moldova	1	118	78	–	–	66.1	57.2–74.0	–	–	–	
*Overall Eastern*	8	12,122	2,036	8.0–78.0	14.2	31.5	19.8–44.6	665.8 (p<0.001)	98.9	0.8–79.0	
***Northern Europe***											p<0.001 (p<0.001)
Denmark	17	474,094	19,350	0.9– 40.1	12.0	6.3	3.7–9.3	22,782.0 (p<0.001)	99.9	0.00–24.1	
Finland	22	749,342	129,062	4.9–36.3	17.3	18.4	16.7–20.2	6,728.1 (p<0.001)	99.7	10.6–27.8	
Iceland	3	168	17	2.3–28.9	9.1	11.0	0.6–29.7	17.5 (p<0.001)	88.6	–	
Ireland	10	8,572	309	1.8–58.4	9.3	18.9	10.0–29.9	376.6 (p<0.001)	97.6	0.0–64.1	
Lithuania	3	3,377	196	2.3–23.6	5.1	8.5	1.4–20.2	45.1 (p<0.001)	95.6	–	
Norway	19	1,332,092	25,092	1.1–63.0	2.0	4.6	3.8–5.5	6,094.2 (p<0.001)	99.7	1.6–8.9	
Sweden	20	7,479,062	74,073	0.2–34.6	1.5	1.8	1.5–2.2	18,241.0 (p<0.001)	99.9	0.6–3.8	
United Kingdom	28	232,214	10,113	1.9–29.8	11.2	11.7	9.4–14.4	6,947.8 (p<0.001)	99.6	1.8–28.6	
*Overall*	122	10,278,921	258,212	0.2–63.0	7.5	8.9	7.9–10.0	365,513.4 (p<0.001)	100.0	1.0–23.4	
***Western Europe***											p<0.001 (p<0.001)
Austria	5	8,897	1,042	4.2–46.0	38.3	27.3	13.0–44.3	796.0 (p<0.001)	99.5	0.0–90.4	
Belgium	2	14,773	614	4.1–11.6	7.9	3.9	3.6–4.3	–	–	–	
France	16	3,109,492	189,173	1.2–43.8	7.5	8.0	5.9–10.4	22,936.1 (p<0.001)	100.0	2.7–17.0	
Germany	18	1,058,242	101,724	3.4–27.6	7.0	7.3	5.1–9.9	61,693.8 (p<0.001)	99.9	0.8–21.3	
Netherlands	4	17,442	3,717	4.5–31.6	14.0	13.9	1.9–34.1	2,340.4 (p<0.001)	99.9	0.0–100.0	
Switzerland	10	3,877	583	10.0–31.7	16.1	17.0	11.3–23.4	120.3 (p<0.001)	92.5	1.7–41.4	
*Overall Western*	55	4,212,723	296,853	1.2–46.0	8.6	10.7	9.5–12.0	73,483.9 (p<0.001)	99.9	3.4–21.4	
**Southern Europe**											p<0.001 (p<0.001)
Croatia	13	88,086	4,676	1.1–23.1	4.7	5.8	3.2–9.2	3,635.5 (p<0.001)	99.7	0.0–24.0	
Cyprus	1	230	45	–	–	19.6	15.0–25.2	–	–	–	
Greece	4	1,122	102	7.6–17.0	9.3	10.0	6.4–14.3	69.6 (p=0.02)	9.9	0.1–31.3	
Italy	32	222,809	13,497	1.7–47.6	11.5	14.5	11.1–18.1	13,663.2 (p<0.001)	99.8	0.9–39.8	
Malta	1	203	43	–	–	21.2	16.1–27.3	–	–	–	
Slovenia	1	450	43	–	–	9.6	7.2–12.6	–	–	–	
Spain	17	756,181	37,786	4.8–39.6	11.4	15.0	11.0–19.4	1,838.4 (p<0.001)	99.1	1.7–37.6	
*Overall Southern*	69	1,069,081	56,192	1.1–47.6	10.7	12.3	10.9–13.9	19,346.8 (p<0.001)	99.6	3.0–28.0	
**OVERALL Europe^8^**	254	15,572,847	613,293	0.2–78.0	9.9	10.9	10.0–11.8	674,742.8 (p<0.001)	100.0	1.4–27.3	

^1^ Q: Cochran’s Q statistic is a measure assessing the existence of heterogeneity in estimates of GDM prevalence.

^2^ I^2^: a measure assessing the percentage of between−study variation that is due to differences in GDM prevalence estimates across studies rather than chance.

^3^ PI: Prediction intervals: estimates the 95% confidence interval in which the true GDM prevalence estimate in a new study is expected to fall.

^4^ Heterogeneity between subgroups using random effects model (fixed effect model).

^5^ Overall pooled GDM prevalence in 4 countries in Europe regardless of the tested population, sample size, and data collection period, using the most updated criteria when GDM ascertained using different criteria in the same population.

^8^ Overall pooled GDM prevalence in ALL Europe

CI, confidence interval calculated using the exact binomial method. GDM: gestational diabetes mellitus.

### Sub-Regional Weighted GDM Prevalence

The highest sub-regional weighted GDM prevalence observed in the three Eastern European countries (31.5%, 95% CI: 19.8–44.6, *I^2^*, 98.9%), followed by 12.3% (95% CI:10.9–13.9, *I^2^*, 99.6%) in Southern Europe, 10.7% (95% CI: 9.5–12.0, *I^2^*, 99.9%) in Western Europe, and 8.9% (95% CI: 7.9–10.0, *I^2^*, 100.0%) in Northern Europe.

### Sub-Group Analysis

The weighted prevalence of GDM was significantly higher in pregnant women ≥30 years old (15.4%, I^2^, 99.8%) compared with 15–29 years old women (7.2%, *I^2^*, 99.6%), in their third (18.4%, I^2^, 99.8%) compared with second trimester (12.5%, *I^2^*, 99.9%) of pregnancy, in obese (23.1%, *I^2^*, 98.3%) and overweight (7.8%, I2, 99.5%) compared with normal weight (3.4%, *I^2^*, 99.4%) pregnant women.

This observation was comparable in the four sub-regions, whenever data was available. In the Northern European sub-region that comprised 48.0% of the GDM prevalence studies and tested 66.0% of the pregnant women in Europe, the weighted prevalence of GDM was 1.86-time higher in pregnant women ≥30 years old (13.4%, I^2^, 99.7%) compared with younger women (7.2%, *I^2^*, 99.7%), 1.83-time higher in the third trimester (18.0%, 95% CI: 10.0–27.7, *I^2^*, 99.8%) compared with the second trimester (9.8%, 95% CI: 7.6–12.2, *I^2^*, 99.9%), 4.2-time and 14.1-time higher in obese (31.1%, 95% CI: 26.5–35.8, *I^2^*, 0.0%) compared with overweight (7.4%) and normal weight (2.2%) women, respectively. In all sub-regions, there was a significant variation (p<0.001) in the weighted GDM prevalence between the used GDM ascertainment guidelines (**Supplementary Table S3**).

### Risk of Bias (RoB)

**Figure 2:**
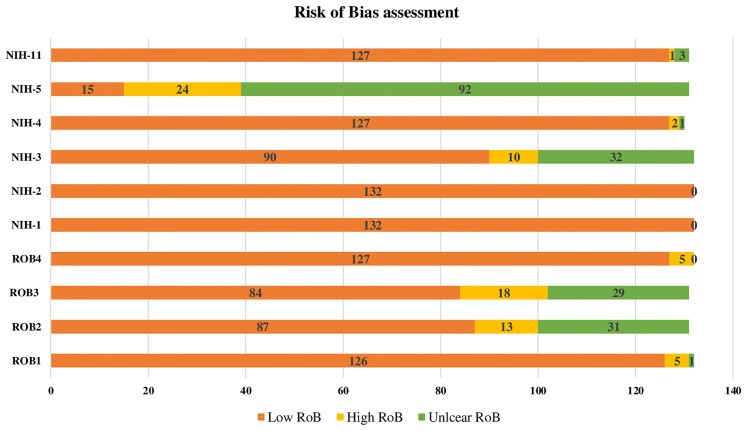
Risk of Bias assesment of the 132 reviewed research reports on GDM. RoB1: GDM ascertainment (1: biological assay/medical records; 2: self-reported; 3: unclear) RoB2: Sampling methodology (1: probability-based ''random, consecutive, or whole population within a specified period of time''; 2: non-probability based; 3: unclear) RoB3: Response rate (1:<80%; 2:80%) RoB4: Precision (1: tested sample size100; 2: tested sample size <100) NIH-1: Was the research question or objective in this paper clearly stated? 1: Low risk of bias (ROB), 2: High ROB, 3: Unclear ROB NIH-2: Was the study population clearly specified and defined? 1: Low ROB, 2: High ROB, 3: Unclear ROB NIH-3: Was the participation rate of eligible persons at least 50%? 1: Low ROB, 2: High ROB, 3: Unclear ROB NIH-3: Was the participation rate of eligible persons at least 50%? 1: Low ROB, 2: High ROB, 3:Unclear ROB prespecified and applied uniformly to ail participants? 1: Low ROB, 2: High ROB, 3: Unclear ROB NIH-5: Was a sample size justification, power description, or variance and effect estimates provided? 1: Low ROB, 2: High ROB, 3: Unclear ROB NIH-11: Were the outcome measures (dependent variables) clearly defined, valid, reliable, and implemented consistently across all study participants? 1: Low ROB, 2: High ROB, 3: Unclear ROB.

The results of the four RoB domains assessed and the six quality of evidence items from NIH are presented in (**Figure 2**). Overall, the RoB and quality of evidence showed a significant low RoB with domains like the study population and research question having 100% of high quality of evidence. Recruitment and outcomes measurement were also rated with high quality of evidence in 97%, while sample size justification was unclear for 70% of the studies. Regarding RoB, GDM ascertainment and precision were low for 4% and 5%, respectively. While the response rate and sampling methodology were considered high for 14% and 10%, respectively (**Figure 2**).

### Publication Bias

Graphically, the funnel plot shows a potential of publication bias and small-study effect (Egger’s test, *p* < 0.001) on the estimated pooled prevalence (**Supplementary Figure S1**).

## Discussion

### Summary of Evidence

This systematic review and meta-analysis research summarizes the prevalence of GDM in Europe based on 133 reports comprising data of 254 single studies reported between 2014 and 2019 in 24 countries. Most of these studies were from Italy and the United Kingdom. The overall estimated prevalence of GDM in the 24 countries from the entire European Region was lower (10.9%, 95% CI: 10.0–11.8, *I^2^*: 100%) than the estimates reported by the International Diabetes Federation (IDF) for 2019 (16.3%) (168) and higher than a previous meta-analysis (5.4%, 95% CI: 3.8–7.8) conducted by Eades and colleagues (22). Differences in the population estimates (and countries) might explain the variation between the reports. IDF has included data of 39 countries and only for women aged 20-45 years old (168) and Eades and colleagues included only 12 countries (22). A descriptive study revising the global GDM prevalence points to Europe as the region with the lowest GDM prevalence with a median of 6.1 (range 1.8%-31.0%) (169), in our study, the median estimate was 9.9 (range 0.2%-78%).

Considering the four sub-regions of Europe, the Eastern region presented the highest GDM prevalence (31.5%, 95% CI: 19.8–44.6, *I^2^*: 98.9%), followed by Southern Europe (12.3%, 95% CI: 10.9–13.9, *I^2^*: 99.6%), Western Europe (10.7%, 95% CI: 9.5–12.0, *I^2^*: 99.9%), and Northern Europe (8.9%, 95% CI: 7.9–10.0, *I^2^*: 100). A review of the literature from 2000-2009 is consistent with these results presenting the lowest GDM prevalence for the European northern or Atlantic seaboard countries in comparison with the Southern or Mediterranean countries (170). The Eastern (and Southern regions were also the two regions with the smallest number of studies included, 4.5% and 27.1% respectively, due to the lack of identified reports from these countries. These results highlight the need for good quality and standardized epidemiological studies in these two regions, not to mention the 25 countries that are not represented in our study. We have assessed full-text studies from some countries like Albania and Portugal that were potentially eligible to be considered but as the GDM ascertainment criteria was not clear, therefore they were excluded for not meeting our criteria.

The Republic of Moldova has the highest GDM prevalence across the entire region (66.1%, 95% CI 19.8-44.6%, I^2^: 98.9%), followed by Poland, Austria, Cyprus, and Malta. Sweden has the lowest GDM prevalence followed by Belgium, Norway, Croatia, and Denmark. The IDF 2019 Diabetes Atlas presents GDM prevalence for 12 countries in the region and their estimated prevalence is within our confidence interval for France, Ireland, Netherlands, Poland, and Sweden (168). For Norway, Spain, and the UK their estimates are higher than ours. These findings may suggest the recent higher reported rates for GDM prevalence compared with previous years as our review comprises data from 2014-2019 and there is just for 2019.

In women with a history of GDM, lifestyle interventions and medical treatment decreased the progression of T2DM by up to 40% (171). Therefore, GDM becomes a public health priority issue as it poses a significant health burden, not only to these pregnancies but also to the future health of both mothers and offspring. In this way, the diagnosis and management of GDM can represent an opportunity for intervention to reduce the burden of T2DM. Strategies to prevent T2DM may incorporate hyperglycemia screening 4 to 12 weeks after the post-partum as recommended by the most recent guidelines from ADA (12).

Differences in the GDM criteria used in the different countries and sub-regions also play an important role in the differences of prevalence reported and most importantly in the heterogeneity of our meta-analysis estimations. It is known that there is a poor consensus and uniformity in the diagnosis of GDM, as our study demonstrates, by having 24 different criteria used. This fact is to be considered as well with the recent criteria updates, specifically from the WHO in 2013. The differences in GDM criteria allied with the different countries’ screening guidelines (e.g., universal GDM screening *vs* screening for women with risk factors) introduce heterogeneity to the meta-analysis and increases the challenge of comparing the prevalence across countries and regions. Standardized studies and policies across the European region would help to tackle the GDM public health burden.

### Strengths, Implications, and Limitations

This study has used a comprehensive search strategy to review all the studies of GDM in Europe at the regional, sub-regional, and national levels. The study includes a huge number of reports and single estimates that were combined. Estimating a weighted GDM prevalence based on a huge number (over 15 million) of tested pregnant women provides the best-precise estimation of the burden of GDM in the included European countries. Additionally, estimating the pooled GDM prevalence among various pregnant women population groups according to age, trimester of GDM diagnosis, maternal body weight, also provides specific estimates in this population group to priorities action and screening strategies. As mentioned above, the range of GDM per country varied widely therefore we are not able to extrapolate the reported GDM prevalence for the European countries not represented in our estimates, the sub-regions itself and even within the countries, as the case of the Republic of Moldova, Iceland, and Malta that are included in our analysis with one single report. Another potential limitation is the lack of or small number of studies from specific countries which might not reflect the reality of the region. Therefore, interpreting the present findings should be exercised in the light of this important potential limitations.

## Conclusions

The overall GDM prevalence in Europe is considerable, particularly for pregnant women in Eastern European countries. Epidemiological studies focusing on GDM and using standardized GDM criteria would be crucial to better estimate the national, sub-regional, and regional GDM of Europe as GDM has serious public health implications for the life of the mothers and newborns. This systematic review and meta-analysis findings highlight these implications and aim to contribute to the vigilant public health awareness campaigns about the risk factors associated with developing GDM in Europe and globally.

## Data Availability Statement

The original contributions presented in the study are included in the article/**Supplementary Material**. Further inquiries can be directed to the corresponding author.

## Ethics Statement

There are no primary data used in this review. There is no need for any ethical approval or an exemption letter according to the United Arab Emirates University-Human Research Ethics Committee.

## Author Contributions 

RHA conceptualized and designed the study. MSP assessed the eligibility of the retrieved citations in the titles/abstracts and full-text screening phases. RHA, NA, and MSP critically assessed the eligible studies and extracted data. RHA and NA performed the analysis. MSP and RB-S wrote the initial draft of the manuscript. All authors contributed to the article and approved the submitted version.

## Funding

This systematic review was funded by the Summer Undergraduate Research Experience (SURE) PLUS-Grant of the United Arab Emirates University, 2017 (Research grant: 31M348). The funder had no role in the study design, collection, analysis, or interpretation of the data, nor in writing and the decision to submit this article for publication.

## Conflict of Interest

The authors declare that the research was conducted in the absence of any commercial or financial relationships that could be construed as a potential conflict of interest.

## Publisher’s Note

All claims expressed in this article are solely those of the authors and do not necessarily represent those of their affiliated organizations, or those of the publisher, the editors and the reviewers. Any product that may be evaluated in this article, or claim that may be made by its manufacturer, is not guaranteed or endorsed by the publisher.

## Abbreviations

ADA, American Diabetes Association; BMI, body mass index; CI, Confidence interval; DM, diabetes mellitus; EVOO, extra virgin olive oil; GDDD, Deutsche Gesellschaft fur gynakologie und Geburtshilfe; GDM, Gestational diabetes mellitus; HAPO, Hyperglycemia and Adverse Pregnancy Outcomes; HIV, Human Immunodeficiency Virus; IADPSG, International Association of the Diabetes and Pregnancy Study Groups; IDF, International Diabetes Federation; NICE, National Institute for Health and Care Excellence; OGTT, oral glucose tolerance test; PDA, Polish Diabetes Association; PECO, Population, Exposure, Comparator and Outcome; PRISMA, Preferred Reporting Items for Systematic Review and Meta-Analysis; RoB, risk of bias; T2DM, type 2 diabetes mellitus; UK, United Kingdom; UN, United Nations; WHO, World Health Organization.
