# Supplementary material for: Gestational Diabetes Mellitus in Europe: A Systematic Review and Meta-Analysis of Prevalence Studies
Source: Front Endocrinol (Lausanne). 2021 Dec 9;12:691033. doi: 10.3389/fendo.2021.691033 (PMC8698118; doi:10.3389/fendo.2021.691033)
Supplement: Supplementary file 2 [file DataSheet_2.docx]

Supplementary Table S2

**PubMed Search Strategy**

((("Diabetes Mellitus"[Mesh] OR "Diabetes Mellitus, Type 2"[Mesh] OR "diabetes type 2"[Mesh] OR "T2DM"[Mesh] OR "diabetes Type II"[Mesh] OR diabetes AND "[Mesh] OR " AND glucose intolerance AND "[Mesh] OR " AND insulin resistance AND "[Mesh] OR " AND Hyperglycemia AND "[Mesh] OR " AND Hypoglycemia AND "[Mesh] OR OR " AND Diabetes Mellitus AND "[Text] OR " AND Diabetes Mellitus, Type 2 AND "[Text] OR " AND diabetes type 2 AND "[Text] OR " AND T2DM AND "[Text] OR " AND diabetes Type II AND "[Text] OR diabetes"[Text] OR "glucose intolerance"[Text] OR "insulin resistance"[Text] OR "Hyperglycemia"[Text] OR "Hypoglycemia"[Text]) AND ("Adolescent"[Mesh] OR "Young Adult"[Mesh] OR "Adult"[Mesh] OR "Middle Aged"[Mesh] OR "teenage"[Mesh] OR "adolescent"[Mesh] OR "young adult"[Mesh] OR "adult"[Mesh] OR "Middle Age"[Mesh] OR "Adolescent"[Text] OR "Young Adult"[Text] OR "Adult"[Text] OR "Middle Aged"[Text] OR "teenage"[Text] OR "adolescent"[Text] OR "young adult"[Text] OR "adult"[Text] OR "Middle Age"[Text]))) AND (((((((((((((((((((((((((((((((((((((((((((((((((((((Albania[Text Word]) OR Andorra[Text Word]) OR Armenia[Text Word]) OR Austria[Text Word]) OR Azerbaijan[Text Word]) OR Belarus[Text Word]) OR Belgium[Text Word]) OR ("Bosnia and Herzegovina"[Text Word])) OR Bulgaria[Text Word]) OR Croatia[Text Word]) OR Cyprus[Text Word]) OR "Czech Republic"[Text Word]) OR Denmark[Text Word]) OR Estonia[Text Word]) OR Finland[Text Word]) OR France[Text Word]) OR Georgia[Text Word]) OR Germany[Text Word]) OR Greece[Text Word]) OR Hungary[Text Word]) OR Iceland[Text Word]) OR Italy[Text Word]) OR Kazakhstan[Text Word]) OR Kosovo[Text Word]) OR Latvia[Text Word]) OR Liechtenstein[Text Word]) OR Lithuania[Text Word]) OR Luxembourg[Text Word]) OR Malta[Text Word]) OR Moldova[Text Word]) OR Monaco[Text Word]) OR Montenegro[Text Word]) OR Netherlands[Text Word]) OR Macedonia[Text Word]) OR Norway[Text Word]) OR Poland[Text Word]) OR Portugal[Text Word]) OR Romania[Text Word]) OR Russia[Text Word]) OR "San Marino"[Text Word]) OR Serbia[Text Word]) OR Slovakia[Text Word]) OR Slovenia[Text Word]) OR Spain[Text Word]) OR Sweden[Text Word]) OR Switzerland[Text Word]) OR Ukraine[Text Word]) OR "United Kingdom"[Text Word]) OR "Vatican City"[Text Word])) OR Europe[MeSH]) AND ("2018/07/12"[PDat] : "3000/12/31"[PDat]) AND Humans[Mesh]) AND ( "2018/07/12"[PDat] : "3000/12/31"[PDat] ) AND Humans[Mesh]
