# Supplementary material for: Gestational Diabetes Mellitus in Europe: A Systematic Review and Meta-Analysis of Prevalence Studies
Source: Front Endocrinol (Lausanne). 2021 Dec 9;12:691033. doi: 10.3389/fendo.2021.691033 (PMC8698118; doi:10.3389/fendo.2021.691033)
Supplement: Supplementary file 3 [file DataSheet_3.docx]

Supplementary Table S3

**Supplementary Table S3**: Sub-regional weighted prevalence of GDM in pregnant women by pregnancy trimester, body mass index, study period and ascertainment methodology.

|  | **Studies** | **Tested sample** | **GDM** | **GDM prevalence** | | | | **Heterogeneity measures** | | | |  |
| --- | --- | --- | --- | --- | --- | --- | --- | --- | --- | --- | --- | --- |
|  |  |  |  | Range (%) | Median  (%) | Weighted (%) | 95% CI | Q (*p*−value)^1^ | *I^2^* (%)^2^ | 95% PI (%)^3^ | *P*−value^4^  (fixed) |  |
| ***Eastern Europe*** |  |  |  |  |  |  |  |  |  |  |  |  |
| **Age** |  |  |  |  |  |  |  |  |  |  |  |  |
| ≥30 years | 5 | 1,042 | 298 | 8.0−78.0 | 13.4 | 34.1 | 8.8−65.8 | 427.0 (*p*<0.001) | 99.1 | 0.0−100.0 | *P*=0.665 (*p*<0.001) |  |
| Unclear age | 3 | 11,080 | 1,738 | 10.1−66.1 | 14.9 | 27.0 | 15.2−40.6 | 175.7 (*p*<0.001) | 98.9 | − |  |  |
| **Trimester** |  |  |  |  |  |  |  |  |  |  |  |  |
| Second | 4 | 1,901 | 450 | 10.1−78.0 | 68.9 | 55.5 | 13.7−93.0 | 612.5 (*p*<0.001) | 99.5 | 0.0−100.0 | *P*=0.044 (*p*<0.001) |  |
| Not reported | 4 | 10,221 | 1,586 | 8.0−14.9 | 13.0 | 12.6 | 8.9−16.8 | 20.1 (*p*<0.001) | 85.1 | 0.7−35.0 |  |  |
| **BMI (No Studies)** |  |  |  |  |  |  |  |  |  |  |  |  |
| **GDM Ascertainment**^9^ |  |  |  |  |  |  |  |  |  |  |  |  |
| WHO 1999 | 1 | 1,493 | 155 | − | − | 10.4 | 8.9−12.0 | − | − | − | *p*<0.001  (*p*<0.001) |  |
| WHO 2013 | 1 | 9,469 | 1,505 | − | − | 15.9 | 15.2−16.6 | − | − | − |  |  |
| IADPSG | 1 | 118 | 78 | − | − | 66.1 | 57.2−74.0 | − | − | − |  |  |
| Polish Gynecological society guidelines | 3 | 752 | 81 | 8.0−13.4 | 12.5 | 11.1 | 7.9−14.7 | 4.3 (*p*=0.114) | 54.0 | − |  |  |
| PDA 2011 | 1 | 145 | 113 | − | − | 77.9 | 70.5−83.9 | − | − | − |  |  |
| PDA 2014 | 1 | 145 | 104 | − | − | 71.7 | 63.9−78.4 | − | − | − |  |  |
| ***Northern Europe*** |  |  |  |  |  |  |  |  |  |  |  |  |
| **Age** |  |  |  |  |  |  |  |  |  |  |  |  |
| 15–29 years | 23 | 417,711 | 12,673 | 0.9−38.0 | 12.0 | 7.2 | 5.6−8.9 | 7,380.9 (*p*<0.001) | 99.7 | 1.2−17.1 | *P*= 0.022  (*P*<0.001) |  |
| ≥30 years | 16 | 129,590 | 19,577 | 1.8−29.6 | 11.3 | 13.4 | 9.5−17.9 | 5811.5 (*p*<0.001) | 99.7 | 1.0−35.9 |  |  |
| Unclear age | 80 | 9,730,834 | 225,904 | 0.2−63.0 | 5.0 | 8.7 | 7.5−9.9 | 315,729 (*p*<0.001) | 100.0 | 1.0−22.4 |  |  |
| Overlapping | 3 | 786 | 58 | 2.3−8.9 | 7.7 | 6.3 | 3.2−10.4 | 7.9 (*p*=0.02) | 74.6 | − |  |  |
| **Trimester** |  |  |  |  |  |  |  |  |  |  |  |  |
| Second | 46 | 562,559 | 29,545 | 1.0−58.1 | 7.5 | 9.8 | 7.6−12.2 | 31,494.9 (*p*<0.001) | 99.9 | 0.2−29.7 | *P*=0.012 (*P*<0.001) |  |
| Third | 8 | 44,405 | 10,678 | 4.2−40.1 | 21.0 | 18.0 | 10.0−27.7 | 3633.5 (*p*<0.001) | 99.8 | 0.0−58.6 |  |  |
| Not reported | 68 | 9,671,957 | 217,989 | 0.2−63.0 | 7.0 | 7.7 | 6.5−9.0 | 293889 (*p*<0.001) | 100.0 | 0.8−20.5 |  |  |
| **BMI** |  |  |  |  |  |  |  |  |  |  |  |  |
| Normal weight | 4 | 81,164 | 1,037 | 1.0−4.9 | 1.8 | 2.2 | 0.3−5.4 | 89.4 (*p*<0.001) | 96.6 | 0.0−25.6 | *P*<0.001 (*P*<0.001) |  |
| Overweight | 4 | 68,447 | 938 | 1.3−15.1 | 9.1 | 7.4 | 0.3−21.1 | 165.2 (*p*<0.001) | 98.2 | 0.0−88.8 |  |  |
| Obese | 3 | 390 | 122 | 28.9−38 | 30.6 | 31.1 | 26.5−35.8 | 1.0 (*p*=0.598) | 0.0 | − |  |  |
| Unclear | 2 | 240 | 50 | 11.6−30.0 | 20.8 | 20.1 | 15.2−25.4 | − | − | − |  |  |
| **GDM Ascertainment**^9^ |  |  |  |  |  |  |  |  |  |  |  |  |
| WHO 1990 | 12 | 143,437 | 5,426 | 2.4−23.8 | 9.0 | 8.7 | 5.7−12.2 | 2,523.7 (*p*<0.001) | 99.6 | 0.4−25.9 | *P*<0.001 (*P*<0.001) |  |
| WHO 2013 | 14 | 48,642 | 11,070 | 1.8−40.1 | 17.5 | 20.0 | 12.3−29.0 | 5,233.7 (*p*<0.001) | 99.8 | 0.0−61.1 |  |  |
| ADA 2007 | 1 | 269 | 47 | − | − | 17.5 | 13.4−22.5 | − | − | − |  |  |
| ADA 2008 | 1 | 251 | 46 | − | − | 18.3 | 14.0−23.6 | − | − | − |  |  |
| Medical records | 55 | 9,777,110 | 232,596 | 0.2−63.0 | 3.0 | 6.1 | 4.9−7.4 | 323198 (*p*<0.001) | 100.0 | 0.3−18.8 |  |  |
| IADPSG | 15 | 4,600 | 1,076 | 1.0−58.1 | 27.1 | 23.7 | 17.8−30.2 | 288.9 (*p*<0.001) | 95.2 | 3.9−52.7 |  |  |
| Carpenter and Coustan | 2 | 280 | 9 | 2.1−4.3 | 3.2 | 3.1 | 1.3−5.6 | − | − | − |  |  |
| Mixed Method | 11 | 261,178 | 4,601 | 1.2−8.9 | 3.6 | 3.2 | 2.5−4.0 | 823.4 (*p*<0.001) | 98.8 | 1.1−6.4 |  |  |
| NICE 2015 | 4 | 9,296 | 1,180 | 11.4−21.0 | 14.3 | 13.3 | 9.0−18.3 | 75.0 (*p*<0.001) | 96.0 | 0.1−41.8 |  |  |
| The Finnish Current Care Guidelines | 3 | 9,660 | 1,722 | 16.5−28.7 | 19.9 | 21.4 | 15.4−28.0 | 59.4 (*p*<0.001) | 96.6 | − |  |  |
| Norwegian Society of Gynecology and Obstetrics | 4 | 24,198 | 439 | 1.1−3.1 | 1.7 | 1.8 | 1.2−2.5 | 38.5 (*p*<0.001) | 92.2 | 0.0−5.9 |  |  |
| ***Western Europe*** |  |  |  |  |  |  |  |  |  |  |  |  |
| **Age** |  |  |  |  |  |  |  |  |  |  |  |  |
| 15–29 years | 4 | 70,870 | 3,335 | 4.2−8.7 | 6.1 | 4.7 | 3.7−5.7 | 50.4 (*p*<0.001) | 94.0 | 1.3−9.5 | *P*<0.001 (*P*<0.001) |  |
| ≥30 years | 18 | 1,533,518 | 144,784 | 4.1−43.8 | 8.2 | 11.0 | 9.2−13.1 | 15,010 (*p*<0.001) | 99.9 | 3.8−21.3 |  |  |
| Unclear age | 31 | 2,515,044 | 143,032 | 1.2−46.0 | 11.0 | 11.7 | 9.9−13.5 | 36389 (*p*<0.001) | 99.9 | 3.8−22.7 |  |  |
| Overlapping | 2 | 93,291 | 5,702 | 5.8−6.5 | 6.1 | 6.1 | 6.0−6.3 | − | − | − |  |  |
| **Trimester** |  |  |  |  |  |  |  |  |  |  |  |  |
| Second | 31 | 1,657,258 | 152,350 | 4.0−46.0 | 13.2 | 12.4 | 10.5−14.4 | 23,881 (*p*<0.001) | 99.9 | 3.8−24.5 | *P=*0.004 (*P*<0.001) |  |
| Not reported | 24 | 2,555,465 | 144,503 | 1.2−44.5 | 7.5 | 9.0 | 7.6−10.6 | 30,586 (*p*<0.001) | 99.9 | 2.8−18.2 |  |  |
| **BMI** |  |  |  |  |  |  |  |  |  |  |  |  |
| Normal weight | 1 | 1,957 | 110 | − | − | 5.6 | 4.7−6.7 | − | − | − | *P*<0.001 (*P*<0.001) |  |
| Overweight | 1 | 548 | 89 | − | − | 16.2 | 13.4−19.6 | − | − | − |  |  |
| Obese | 1 | 305 | 65 | − | − | 21.3 | 17.1−26.2 | − | − | − |  |  |
| **GDM Ascertainment**^9^ |  |  |  |  |  |  |  |  |  |  |  |  |
| WHO 1999 | 1 | 2,889 | 129 | − | − | 4.5 | 3.8−5.3 | − | − | − | *P*<0.001 (*P*<0.001) |  |
| WHO 2013 | 2 | 13,935 | 3,917 | 16.8−31.6 | 24.2 | 27.8 | 27.1−28.6 | − | − | − |  |  |
| ADA 2003/2004 | 1 | 188 | 43 | − | − | 22.9 | 17.4−29.4 | − | − | − |  |  |
| ADA 2016 | 1 | 218 | 32 | − | − | 14.7 | 10.6−20.0 | − | − | − |  |  |
| Mixed Method | 11 | 89,027 | 3,327 | 3.4−28.5 | 14.4 | 10.3 | 7.1−13.9 | 327.3 (*p*<0.001) | 96.9 | 1.6−23.9 |  |  |
| Medical records | 19 | 3,826,593 | 273,021 | 1.2−31.7 | 7.2 | 7.9 | 6.2−9.8 | 63,375 (*p*<0.001) | 100.0 | 1.6−18.1 |  |  |
| IADPSG | 14 | 260,406 | 15,452 | 4.0−44.5 | 9.7 | 12.8 | 11.0−14.8 | 2,140.8 (*p*<0.001) | 99.4 | 6.1−21.5 |  |  |
| Carpenter and Coustan | 4 | 19,299 | 874 | 4.1−7.1 | 5.9 | 5.5 | 4.2−7.0 | 26.8 (*p*<0.001) | 88.8 | 1.0−13.4 |  |  |
| German Diabetes Association | 1 | 105 | 29 | − | − | 27.6 | 20.0−36.8 | − | − | − |  |  |
| GDDD | 1 | 63 | 29 | − | − | 4.6 | 34.3−58.2 | − | − | − |  |  |
| ***Southern Europe*** |  |  |  |  |  |  |  |  |  |  |  |  |
| **Age** |  |  |  |  |  |  |  |  |  |  |  |  |
| 15–29 years | 4 | 1,122 | 102 | 7.6−17 | 9.3 | 10.0 | 6.4−14.3 | 9.9 (*p*=0.02) | 69.6 | 0.1−31.3 | *P*<0.001 (*P*<0.001) |  |
| ≥30 years | 28 | 51,218 | 6,711 | 2.8−47.6 | 17.4 | 17.6 | 13.3−22.2 | 3,970 (*p*<0.001) | 99.3 | 1.0−47.4 |  |  |
| Unclear age | 36 | 930,570 | 47,458 | 1.1−31.8 | 9.9 | 9.5 | 7.9−11.3 | 10,171(*p*<0.001) | 99.7 | 1.7−22.4 |  |  |
| Overlapping | 1 | 86,171 | 1,921 | − | − | 2.2 | 2.1−2.3 | − | − | − |  |  |
| **Trimester** |  |  |  |  |  |  |  |  |  |  |  |  |
| Second | 46 | 164,376 | 10,456 | 1.1−39.9.5 | 11.1 | 12.9 | 10.3−15.8 | 10,444 (*p*<0.001) | 99.6 | 0.8−36.7 | *P*<0.001 (*P*<0.001) |  |
| Third | 2 | 9,292 | 1,900 | 17.8−23.1 | 20.5 | 20.4 | 19.6−21.2 | − | − | − |  |  |
| Not reported | 21 | 895,413 | 43,836 | 1.7−47.6 | 8.4 | 10.8 | 8.8−13.0 | 6563.1 (*p*<0.001) | 99.7 | 2.8−23.1 |  |  |
| **BMI** |  |  |  |  |  |  |  |  |  |  |  |  |
| Underweight | 3 | 1,038 | 19 | 1.1−10.7 | 1.5 | 2.5 | 0.4−5.9 | 11.3 (*p=*0.004) | 82.2 | − | *P*<0.001 (*P*<0.001) |  |
| Normal weight | 4 | 38,726 | 1,531 | 1.2−7.9 | 4.2 | 3.9 | 1.7−6.9 | 438.4 (*p*<0.001) | 99.3 | 0.0−25.3 |  |  |
| Overweight | 4 | 14,853 | 1,025 | 2.8−13.6 | 6.2 | 6.7 | 2.4−12.9 | 461.8 (*p*<0.001) | 99.4 | 0.0−49.3 |  |  |
| Obese | 6 | 6,058 | 819 | 4.7−47.6 | 19.4 | 19.7 | 10.7−30.5 | 427.5 (*p*<0.001) | 98.8 | 0.0−63.8 |  |  |
| **GDM Ascertainment**^9^ |  |  |  |  |  |  |  |  |  |  |  |  |
| WHO 1999 | 4 | 17,079 | 357 | 1.1−4.7 | 2.0 | 2.3 | 1.0−4.0 | 101.3 (*p*<0.001) | 97.0 | 0.0−14.5 | *P*<0.001  (*P*<0.001) |  |
| WHO 2006 | 1 | 203 | 43 | − | − | 21.2 | 16.1−27.3 | − | − | − |  |  |
| ADA 2011 | 2 | 197 | 39 | 11.6−27.5 | 19.5 | 19.2 | 13.9−25.0 | − | − | − |  |  |
| ADA 2014 | 3 | 56,211 | 1,654 | 2.7−8.2 | 5.7 | 5.1 | 2.5−8.4 | 92.1 (*p*<0.001) | 97.8 | − |  |  |
| IADPSG | 21 | 87,233 | 6,523 | 1.5−39.6 | 17.1 | 14.8 | 10.1−20.2 | 7,244 (*p*<0.001) | 99.7 | 0.1−46.6 |  |  |
| Self–reported | 1 | 450 | 43 | − | − | 9.6 | 7.2−12.6 | − | − | − |  |  |
| Medical records | 15 | 879,188 | 42,889 | 2.3−47.6 | 9.2 | 11.8 | 9.5−14.4 | 5,199 (*p*<0.001) | 99.7 | 3.5−24.1 |  |  |
| Mixed Method | 2 | 430 | 30 | 6.1−7.6 | 6.8 | 7.0 | 4.7−9.6 | − | − | − |  |  |
| Carpenter and Coustan | 6 | 2,872 | 287 | 7.6−17.0 | 10.1 | 10.0 | 8.0−12.1 | 13.4 (*p=*0.020) | 62.6 | 4.7−16.8 |  |  |
| National Diabetes Data Group | 8 | 7,021 | 769 | 7.4−29.9 | 12.4 | 14.9 | 10.5−19.9 | 78.2 (p<0.001) | 91.0 | 2.7−34.1 |  |  |
| NICE 2015 | 1 | 4,646 | 826 | − | − | 17.8 | 16.7−18.9 | − | − | − |  |  |
| Italian Minister Guidelines | 1 | 5,473 | 1,559 | − | − | 28.5 | 27.3−29.7 | − | − | − |  |  |
| Italian National Guidelines | 1 | 1,338 | 534 | − | − | 39.9 | 37.3−42.6 | − | − | − |  |  |
| HAPO study guidelines | 2 | 6,407 | 593 | 6.6−12.0 | 9.3 | 9.1 | 8.4−9.8 | − | − | − |  |  |
| Spanish society for gynecology and obstetrics | 1 | 333 | 46 | − | − | 13.8 | 10.5−17.9 | − | − | − |  |  |
| **Regardless of the subregion** |  |  |  |  |  |  |  |  |  |  |  |  |
| **Age** |  |  |  |  |  |  |  |  |  |  |  |  |
| 15–29 years | 31 | 489,703 | 16,110 | 0.9−38 | 10.1 | 7.2 | 5.9−8.7 | 8,254.2 (*p*<0.001) | 99.6 | 1.5−16.5 | *P*<0.001  (*P*<0.001) |  |
| ≥30 years | 67 | 1,715,368 | 171,370 | 1.8−78 | 12.5 | 15.4 | 14.0−16.9 | 28,604.1 (*p*<0.001) | 99.8 | 5.8−28.5 |  |  |
| Unclear age | 150 | 13,187,528 | 418,132 | 0.2−66.1 | 8.4 | 9.8 | 8.9−10.8 | 476,017 (*p*<0.001) | 100.0 | 1.4−24.4 |  |  |
| Overlapping | 6 | 180,248 | 7,681 | 2.3−8.9 | 6.1 | 5.3 | 3.1−7.9 | 1,831.1 (*p*<0.001) | 99.7 | 0.1−16.9 |  |  |
| **Trimester** |  |  |  |  |  |  |  |  |  |  |  |  |
| Second | 127 | 2,386,094 | 192,801 | 1.0−78.0 | 10.0 | 12.5 | 11.3−13.8 | 85,164.4 (*p*<0.001) | 99.9 | 2.2−29.2 | *P*<0.001  (*P*<0.001) |  |
| Third | 10 | 53,697 | 12,578 | 4.2−40.1 | 20.5 | 18.4 | 11.9−26.0 | 3,700.0 (*p*<0.001) | 99.8 | 0.8−51.3 |  |  |
| Not reported | 117 | 13,133,056 | 407,914 | 0.2−63.0 | 8.0 | 8.7 | 7.7−9.7 | 445,840 (*p*<0.001) | 100.0 | 1.1−22.3 |  |  |
| **BMI** |  |  |  |  |  |  |  |  |  |  |  |  |
| Underweight | 3 | 1,038 | 19 | 1.1−10.7 | 1.5 | 2.5 | 0.4−5.9 | 11.3 (*p*=0.004) | 82.2 | − | *P*<0.001  (*P*<0.001) |  |
| Normal weight | 9 | 121,847 | 2,678 | 1.0−7.9 | 2.9 | 3.4 | 1.8−5.4 | 1,298.6 (*p*<0.001) | 99.4 | 0.0−12.6 |  |  |
| Overweight | 9 | 83,848 | 2,052 | 1.3−16.2 | 9.0 | 7.8 | 3.9−13.0 | 1,717.0 (*p*<0.001) | 99.5 | 0.0−31.6 |  |  |
| Obese | 10 | 6,753 | 1,006 | 4.7−47.6 | 25.4 | 23.1 | 15.1−32.1 | 524.2 (*p*<0.001) | 98.3 | 1.2−59.7 |  |  |
| Unclear | 2 | 240 | 50 | 11.6−30.0 | 20.8 | 20.1 | 15.2−25.4 | − | − | − |  |  |
| **GDM Ascertainment**^9^ |  |  |  |  |  |  |  |  |  |  | *P*<0.001  (*P*<0.001) |  |
| WHO 1999 | 18 | 164,898 | 6,067 | 1.1−23.8 | 5.0 | 6.7 | 4.8−8.9 | 2,888.5 (*p*<0.001) | 99.4 | 0.5−19.3 |  |  |
| WHO 2006 | 1 | 203 | 43 | − | − | 21.2 | 16.1−27.3 | − | − | − |  |  |
| WHO 2013 | 17 | 72,046 | 16,492 | 1.8−40.1 | 16.8 | 20.1 | 14.1−26.8 | 6,047.8 (*p*<0.001) | 99.7 | 1.0−52.9 |  |  |
| ADA 2003/2004 | 1 | 188 | 43 | − | − | 22.9 | 17.4−29.4 | − | − | − |  |  |
| ADA 2007 | 1 | 269 | 47 | − | − | 17.5 | 13.4−22.5 | − | − | − |  |  |
| ADA 2008 | 1 | 251 | 46 | − | − | 18.3 | 14.0−23.6 | − | − | − |  |  |
| ADA 2011 | 2 | 197 | 39 | 11.6−27.5 | 19.5 | 19.2 | 13.9−25.0 | − | − | − |  |  |
| ADA 2014 | 3 | 56,211 | 1,654 | 2.7−8.2 | 5.7 | 5.1 | 2.5−8.4 | 92.1 (*p*<0.001) | 97.8 | − |  |  |
| ADA 2016 | 1 | 218 | 32 | − | − | 14.7 | 10.6−20.0 | − | − | − |  |  |
| IADPSG | 51 | 352,357 | 23,129 | 1.0−66.1 | 18.4 | 17.1 | 15.1−19.3 | 11,059.5 (*p*<0.001) | 99.5 | 5.2−33.9 |  |  |
| Carpenter and Coustan | 12 | 22,451 | 1,170 | 2.1−17.0 | 7.3 | 7.2 | 5.5−9.1 | 158.9 (*p*<0.001) | 93.1 | 1.9−15.2 |  |  |
| Medical records | 89 | 14,482,891 | 548,506 | 0.2−63.0 | 6.1 | 7.4 | 6.3−8.6 | 601,801 (*p*<0.001) | 100.0 | 0.3−21.9 |  |  |
| Mixed methods | 24 | 350,635 | 7,958 | 1.2−28.5 | 6.8 | 5.0 | 4.1−6.0 | 2,206.6 (*p*<0.001) | 99.0 | 1.5−10.0 |  |  |
| NICE 2015 | 5 | 13,942 | 2,006 | 11.4−21.0 | 16.5 | 14.2 | 10.5−18.4 | 141.0 (*p*<0.001) | 97.2 | 2.7−32.4 |  |  |
| Self–reported | 1 | 450 | 43 | − | − | 9.6 | 7.2−12.6 | − | − | − |  |  |
| National Diabetes Data Group | 8 | 7,021 | 769 | 7.4−29.9 | 12.4 | 14.9 | 10.5−19.9 | 78.2 (p<0.001) | 91.0 | 2.7−34.1 |  |  |
| Polish Gynecological society guidelines | 3 | 752 | 81 | 8.0−13.4 | 12.5 | 11.1 | 7.9−14.7 | 4.3 (*p*=0.114) | 54.0 | − |  |  |
| PDA 2011 | 1 | 145 | 113 | − | − | 77.9 | 70.5−83.9 | − | − | − |  |  |
| PDA 2014 | 1 | 145 | 104 | − | − | 71.7 | 63.9−78.4 | − | − | − |  |  |
| The Finnish Current Care Guidelines | 3 | 9,660 | 1,722 | 16.5−28.7 | 19.9 | 21.4 | 15.4−28.0 | 59.4 (*p*<0.001) | 96.6 | − |  |  |
| Norwegian Society of Gynecology and Obstetrics | 4 | 24,198 | 439 | 1.1−3.1 | 1.7 | 1.8 | 1.2−2.5 | 38.5 (*p*<0.001) | 92.2 | 0.0−5.9 |  |  |
| German Diabetes Association | 1 | 105 | 29 | − | − | 27.6 | 20.0−36.8 | − | − | − |  |  |
| GDDD | 1 | 63 | 29 | − | − | 4.6 | 34.3−58.2 | − | − | − |  |  |
| Italian Minister Guidelines | 1 | 5,473 | 1,559 | − | − | 28.5 | 27.3−29.7 | − | − | − |  |  |
| Italian National Guidelines | 1 | 1,338 | 534 | − | − | 39.9 | 37.3−42.6 | − | − | − |  |  |
| HAPO study guidelines | 2 | 6,407 | 593 | 6.6−12.0 | 9.3 | 9.1 | 8.4−9.8 | − | − | − |  |  |
| Spanish society for gynecology and obstetrics | 1 | 333 | 46 | − | − | 13.8 | 10.5−17.9 | − | − | − |  |  |

^1^ Q: Cochran’s Q statistic is a measure assessing the existence of heterogeneity in estimates of GDM prevalence.

^2^ *I*^2^: a measure assessing the percentage of between−study variation that is due to differences in GDM prevalence estimates across studies rather than chance.

^3^ Prediction intervals: estimates the 95% confidence interval in which the true GDM prevalence estimate in a new study is expected to fall.

^4^ Overall pooled estimate regardless of the tested population, sample size, and data collection period, using the most updated criteria when GDM ascertained using different criteria in the same population

CI, confidence interval calculated using the exact binomial method.
ADA: American Diabetes Association; BMI: body mass index; GDM: gestational diabetes mellitus; HAPO: Hyperglycaemia and Adverse Pregnancy Outcomes; IADPSG: International Association of Diabetes in Pregnancy Studies Group; NICE: National Institute for Health and Care Excellence; WHO: World Health Organization.
