# Supplementary material for: Gestational Diabetes Mellitus in Europe: A Systematic Review and Meta-Analysis of Prevalence Studies
Source: Front Endocrinol (Lausanne). 2021 Dec 9;12:691033. doi: 10.3389/fendo.2021.691033 (PMC8698118; doi:10.3389/fendo.2021.691033)
Supplement: Supplementary file 4 [file DataSheet_4.docx]

Supplementary material S4

**Figure S1**. Funnel plot examining the small-study effects on the pooled GDM prevalence in pregnant women. The estimated bias coefficient is 4.39 with a standard error of 0.82, indicating a *p* value of <0.001.
